# Dietary Tryptophan Supplementation Attenuates Lipopolysaccharide-Induced Acute Lung Injury in a Murine Model of Colitis

**DOI:** 10.3390/nu18132042

**Published:** 2026-06-23

**Authors:** Hsiao-Ching Lai, Hitoshi Shirakawa, Afifah Zahra Agista, Yi-Ping Hao, Suh-Ching Yang, Ming-Tsan Lin, Sung-Ling Yeh, Chiu-Li Yeh

**Affiliations:** 1 School of Nutrition and Health Sciences, College of Nutrition, Taipei Medical University, Taipei 11031, Taiwan; ma07111009@tmu.edu.tw (H.-C.L.); ma07113006@tmu.edu.tw (Y.-P.H.); sokei@tmu.edu.tw (S.-C.Y.); 2 Research Center of Geriatric Nutrition, College of Nutrition, Taipei Medical University, Taipei 11031, Taiwan; 3 Laboratory of Nutrition, Graduate School of Agricultural Science, Tohoku University, Sendai 980-8857, Japan; shirakah@tohoku.ac.jp (H.S.); afifah.zahra.agista.b2@tohoku.ac.jp (A.Z.A.); 4 Department of Surgery, National Taiwan University Hospital, National Taiwan University College of Medicine, Taipei 10048, Taiwan; linmt@ntu.edu.tw (M.-T.L.); sangling@tmu.edu.tw (S.-L.Y.); 5 Research Center for Digestive Medicine, Taipei Medical University Hospital, Taipei 11031, Taiwan

**Keywords:** aryl hydrocarbon receptor, interleukin-22, toll-like receptor 4, tight junction protein, capillary–epithelial barrier integrity

## Abstract

**Objectives**: Inflammatory bowel disease (IBD) is associated with extraintestinal comorbidities, and lung diseases are widespread manifestations. Respiratory bacterial insult is a common illness that results in acute lung injury (ALI) in critical patients. IBD concurrence with respiratory infection may further exacerbate lung injury. Tryptophan (Try), an essential amino acid, is processed by gut microbiota and produces aryl hydrocarbon receptor (AhR) ligands. These ligands can activate the AhR pathway that exerts anti-inflammatory properties and provides protection against mucosal barrier injury. This study investigated the effects of dietary Try on lipopolysaccharide (LPS)-stimulated ALI in mice with colitis induced by dextran sodium sulfate (DSS). **Methods**: Mice with colitis were allocated to four groups: (1) ND-Sal: normal diet + DSS + intratracheal saline injection; (2) ND-LPS: normal diet + DSS + intratracheal LPS injection; (3) TD-Sal: Try diet + DSS + intratracheal saline injection; (4) TD-LPS: Try diet + DSS + intratracheal LPS injection. Mice were sacrificed 24 h after the intratracheal injection. **Results**: Results showed that colitis resulted in a high disease activity index. Following induction of ALI in colitis mice, neutrophil populations and inflammatory cytokine levels in bronchoalveolar lavage fluid increased. Gene expression levels associated with toll-like receptor (TLR)4/nuclear factor (NF)-κB signaling were upregulated, and tight junction proteins decreased in the lungs. Dietary Try supplementation decreased circulating LPS levels, suppressed pulmonary TLR4/NF-κB signaling, upregulated AhR/interleukin-22 expression, attenuated oxidative stress and improved the capillary–epithelial barrier integrity in DSS-treated mice. **Conclusions**: These findings imply that Try may have potential therapeutic significance in bacterial-induced ALI in a colitis condition.

## 1. Introduction

Inflammatory bowel disease (IBD) is a multifactorial disorder that leads to excessive inflammation and mucosal barrier damage of the gastrointestinal (GI) tract. Previous studies reported that IBD is associated with various comorbidities that arise at extraintestinal sites, and secondary lung diseases have become widespread manifestations in IBD patients [1,2]. The intestinal–pulmonary crosstalk may drive these associations. The GI lumen and respiratory tract have many structural similarities, and similar immune and inflammatory components of these two organs may cause common pathological changes in intestinal and pulmonary mucosal diseases [3]. Previous evidence indicated that microbial dysbiosis and increased intestinal permeability are closely related to IBD-induced lung injury [3]. Lipopolysaccharide (LPS) leaking from the GI tract triggers systemic inflammatory responses and oxidative stress that damage lung tissues [1,2]. Patients with IBD are at increased risk of developing lung disease, and IBD comorbidity with a respiratory infection may further exacerbate lung injury. Respiratory bacterial infections are common illnesses that affect different parts of the respiratory system. Among a variety of symptoms and conditions, acute lung injury (ALI) and its severe form, acute respiratory distress syndrome (ARDS), are highly associated with mortality in critically ill patients [4,5]. Treating respiratory infections in IBD patients is an important concern that warrants investigation.

Tryptophan (Try) is an essential amino acid that is able to produce a number of aryl hydrocarbon receptor (AhR) ligands. The kynurenine metabolic pathway is the most important, because more than 95% of dietary Try is catabolized by this pathway. The diverse bioactive metabolites derived from Try metabolism all possess AhR affinity [6]. AhR ligands activate the AhR that exerts anti-inflammatory properties and provides protection against mucosal barrier injury [7,8,9]. The AhR is highly active in pulmonary endothelial and alveolar epithelial cells. A previous study reported that administration of an AhR agonist preserves alveolar integrity in ALI [10]. Also, AhR signaling in the endothelium was found to be an active mechanism for protecting against lung damage during viral infections [11].

In this study, we investigated the effects of dietary Try on LPS-induced ALI in mice with dextran sodium sulfate (DSS)-induced colitis. LPS is a component of the outer membrane of Gram-negative bacteria, and LPS-induced lung injury is one of the most common rodent models of ALI and ARDS [12,13]. DSS is a chemical that is commonly used in rodent models to induce intestinal inflammation and epithelial barrier destruction [14]. This animal model is thought to resemble pathological changes in ulcerative colitis [15]. We created a colitis mouse model to evaluate the effects of dietary Try supplementation on LPS-induced pulmonary inflammation and capillary-epithelial barrier disruption. It was hypothesized that supplementation with dietary Try would upregulate pulmonary AhR expression, alleviate inflammation and barrier dysfunction, and mitigate LPS-induced lung injury in DSS-induced colitis.

## 2. Materials and Methods

### 2.1. Animals

Six-week-old C57BL/6J male mice from the National Animal Center (Taipei, Taiwan) were raised in the Laboratory Animal Center at Taipei Medical University (TMU; Taipei, Taiwan). The animals were maintained in a humidity (50% ± 10%) and temperature-controlled (22 ± 2 °C) room with a 12 h light–dark cycle. During the 7-day acclimation period, rodent chow and water were provided ad libitum. Care of the laboratory animals was in accordance with the Guide for the Care and Use of Laboratory Animals, and experimental protocols were approved by the Animal Care and Use Committee of TMU (LAC-2019-0564).

### 2.2. Experimental Procedures

After 1-week’s acclimation, mice with comparable body weights (BWs) were divided into the normal control group (NC, *n* = 16) and the Try control group (TC, *n* = 16) using a body-weight-matched allocation approach to ensure comparable baseline BWs between groups. Mice in the NC group were fed the AIN-93M diet (02960397-CF, MP Biomedicals, Irvine, CA, USA), while mice in the TC group were provided with the AIN-93M-based diet in which 0.5% (*w*/*w*) Try was substituted for an equivalent portion of cornstarch. The experimental diets were adopted from a previous study [16]. Diet compositions are illustrated in Supplementary Table S1. After feeding the respective diets for 3 weeks, feces of three mice in each of the NC and TC groups were collected for Try metabolite analysis. The compositions of the experimental diets are presented in Supplementary Table S1. No significant difference in BW was observed between the NC and TC groups at the end of the 3-week dietary intervention. Mice in the NC and TC groups were stratified according to body weight and then randomly assigned to the following four groups using a random number generator to maintain comparable body weights among groups before DSS administration, with 8 mice in each group. All saline or LPS treatments were administered by intratracheal instillation: (1) ND-Sal: normal diet + DSS + saline; (2) ND-LPS: normal diet + DSS + LPS (2 µg/g BW, SI-L2630, Sigma-Aldrich, St. Louis, MO, USA); (3) TD-Sal: Try diet + DSS + saline; and (4) TD-LPS: Try diet + DSS + LPS. Then all mice were given 2% (*w*/*v*) DSS (MW 40 kDa; cat. no. 160110, MP Biomedicals) in distilled water for 5 days, followed by distilled water for a further 5 days for recovery, as previously described [17]. The respective diets were sustained throughout the experimental period. Food intake, water consumption, and BW changes were monitored and recorded daily. Fecal samples were collected before and during the colitis induction period. The disease activity index (DAI) was calculated based on a combination of weight loss, stool consistency, and fecal bleeding, as modified from previous studies [18,19]. The area under the curve (AUC) of the consecutive line during the DSS-treated period was calculated. At the end of the recovery period, the mice were subjected to intratracheal instillation of saline or LPS according to their assigned groups to investigate the influence of Try on LPS-induced ALI in colitis. Before the intratracheal instillation procedure, the mice were anesthetized with an intraperitoneal injection of zoletil (25 mg/kg BW, Virbac, Carros, France) and rompun (10 mg/kg BW, Bayer, Leverkusen, Germany). Intratracheal administration was performed as previously described with minor modifications [20]. A mouse was positioned supine at a 45° angle. The needle of the Micro Sprayer was inserted through the oropharynx and advanced to the tracheal bifurcation. Within 1 s, the plunger flange of the Micro Sprayer was quickly pushed forward, converting the 50 μL of LPS-free saline or LPS-saline solution into an aerosol that was sprayed directly into the lungs. For rehydration, 100 µL of sterile saline was subcutaneously injected. After the procedure, the mice were placed on a warming pad to maintain body temperature until full recovery from anesthesia. At 24 h after intratracheal administration of saline or LPS, the mice were placed under deep anesthesia through intraperitoneal injection using the same anesthetic regimen, zoletil (25 mg/kg BW) and rompun (10 mg/kg BW), before euthanasia. Whole blood, bronchoalveolar lavage fluid (BALF), and lung tissues were then collected for analysis. Whole blood, bronchoalveolar lavage fluid (BALF), and lung tissues were then collected for analysis. No animals or data points were excluded from the analysis. Blood samples were centrifuged at 3000 rpm and 4 °C for 10 min to obtain plasma. Specimens were stored at −80 °C for further analysis. BALF was isolated by inserting a catheter into the trachea and gently flushing with 1 mL ice-cold phosphate-buffered saline (PBS). The flushing fluid was collected and centrifuged at 4 °C and 1000 rpm. The supernatant was collected for further analysis, and the cellular precipitate was resuspended with staining buffer for a flow cytometric analysis. A small portion of the lungs was fixed in 10% formalin for a histopathological examination, and the remaining parts were stored at −80 °C until being further analyzed.

### 2.3. Measurements of Plasma LPS and Cytokine Levels

LPS concentrations were measured using an enzyme-linked immunosorbent assay (ELISA) kit (cat. no. EU3126, FineTest^®^, Wuhan, Hubei, China). Plasma cytokines were measured using the ProcartaPlex™ Mouse Th1/Th2/Th9/Th17/Th22/Treg Cytokine Panel, 17-plex, utilizing high-throughput liquid protein chips (cat. no. EPX170-26087-901, Thermo Scientific, Waltham, MA, USA). This panel is capable of quantifying a number of cytokines. However, only interleukin (IL)-2, IL-6, IL-22, and tumor necrosis factor (TNF)-α were detected in this study. The samples were measured according to the manufacturer’s instructions.

### 2.4. Populations of Macrophages and Neutrophils in BALF

Leukocytes isolated from the BALF were stained with a LIVE/DEAD^TM^ Fixable Near-IR (infrared) Dead Cell Stain kit (cat. no. L34975, Invitrogen, Carlsbad, CA, USA) to exclude dead cells. Living cells were stained with CD45.2-Pacific Blue (PB, cat. no. 109820, Biolegend, San Diego, CA, USA), F4/80-Brilliant Violet 711 (BV711, cat. no. 123147, Biolegend), and Ly-6G-fluorescein (FITC, cat. no. 127605, Biolegend). Different cell types were recognized based on the scatter characteristics and expressions of specific markers. Macrophages were identified as CD45.2^+^/F4/80^+^, and neutrophils were recognized as CD45.2^+^/Ly-6G^+^. Total counts and percentages of neutrophils and macrophages were calculated according to gating of 50,000 leukocytes.

### 2.5. Concentrations of Inflammatory Mediators in BALF

Inflammatory cytokines, including IL-1β (cat. no. MLB00C), IL-6 (cat. no. M6000B), and TNF-α (cat. no. MTA00B) levels were quantified using commercial ELISA kits (R&D Systems, Minneapolis, MN, USA). Concentrations of chemotactic proteins of the chemokines C-X-C motif chemokine ligand (CXCL)1/keratinocyte-derived chemokine (KC) (cat. no. MKC00B) and CXCL2/macrophage inflammatory protein (MIP)-2 (cat. no. MM200) were also analyzed using ELISA kits (R&D Systems). These mediators were corrected based on the protein content in the BALF. Protein levels were analyzed with a commercial kit (Bio-Rad, Hercules, CA, USA).

### 2.6. Messenger (m)RNA Extraction and Analysis of a Real-Time Reverse-Transcription Quantitative Polymerase Chain Reaction (RT-qPCR) in Lung Tissues

Total RNA from lung tissues was isolated, and pellets were used for the analysis. All processing procedures and experimental conditions followed a study we performed previously [21]. The genes we measured were TLR4 pathway-associated components including *TLR4*, *MyD88*, and nuclear factor (*NF*)-*κB*, the *AhR/IL-22/IL-22R* pathway and inflammatory cytokines (*IL-1β*, *IL-6*, and *TNF-α*). Primers were obtained from the GenBank database (National Center for Biotechnology Information, Bethesda, MD, USA), which was provided by Mission Biotech (Taipei, Taiwan). Sequences of primers are presented in Supplemental Table S2. The relative mRNA expression was calculated through the comparative cycle threshold (CT) (2^−ΔΔCt^) method and normalized to mouse 18S ribosomal RNA. The SS group was treated as a calibrator. The fold changes in mRNA expression levels of the SD, PS, and PD groups were calculated relative to the SS group.

### 2.7. Tight Junction (TJ) Protein Levels in Lung Tissues

Thirty milligrams of lung tissue was homogenized in Tissue Protein Extraction Reagent (cat. no. 78510, T-PER™, ThermoFisher Scientific, Waltham, MA, USA) with a protease and phosphatase inhibitor (cat. no. 78442, ThermoFisher Scientific) to extract total protein. Homogenates were centrifuged at 4 °C and 12,000 rpm for 10 min, and supernatants were collected for analysis. Zona occludens (ZO)-1 (cat. no. EM1410, FineTest^®^, Wuhan, Hubei, China) and claudin-5 (cat. no. EM1956, FineTest^®^) were all analyzed using ELISA kits (FineTest^®^). Data are expressed as pg/mg protein, and protein concentrations were measured with a commercial kit (Bio-Rad).

### 2.8. Analysis of Lipid Peroxide Levels in Lung Tissues

Malondialdehyde (MDA) and 4-hydroxynonenal (4-HNE) are the main polyunsaturated fatty acid lipid peroxidation products [22,23]. MDA reacts with thiobarbituric acid to form thiobarbituric acid-reactive substances (TBARSs). TBARS concentrations in biological samples are considered biomarkers of oxidative stress [23]. Lung tissues were homogenized and supernatants were collected as mentioned above. TBARS were analyzed with a commercial assay kit (cat. no. 700870, Cayman, Ann Arbor, MI, USA) as previously described [24]. 4-HNE levels were measured using an Assay Kit (cat. no. ab238538, Abcam, Cambridge, London, UK). TBARS and 4-HNE levels were expressed based on the amount of protein in the homogenates. Protein concentrations were measured with a Bradford protein assay reagent kit (Bio-Rad).

### 2.9. Histopathology of Colon and Lung Tissues

Histopathological examinations of colon and lung tissues were conducted by a pathologist who was blinded to the study. Formalin-fixed specimens were stained with hematoxylin and eosin (H&E) to examine tissue morphology. Procedures were performed as previously described [25]. Digital images of each section were captured at 200× magnification. The colonic injury score was calculated as the sum of three histological features: (1) loss of epithelium, (2) inflammatory cell infiltration, and (3) epithelial injury/ulceration, based on previously described scoring systems for DSS-induced colitis with minor modifications [26]. Loss of epithelium was scored as 0–3 points (0 indicates no loss of epithelium, 1 indicates 0–20% loss of epithelium, 2 indicates 20–30% loss of epithelium, and 3 indicates >30% loss of epithelium). Inflammatory cell infiltration was scored as 0–3 points (0 indicates no lesion, 1 indicates mucosal infiltration, 2 indicates mucosal and submucosal infiltration, and 3 indicates mucosal, submucosal, and transmural infiltration). Epithelial injury/ulceration was scored as 0–3 points (0 indicates no lesion, 1 indicates basal one-third to two-thirds damage involving only the crypts, 2 indicates that only the surface epithelium remained intact, and 3 indicates complete loss of normal mucosal tissue). The total colonic injury score ranged from 0 to 9. The lung injury score was calculated as the sum of three histological features: (1) alveolar wall thickness, (2) inflammatory leukocyte infiltration into the perivascular wall, and (3) pulmonary edema. Each feature was scored as 0–3 points (0 indicates no lesions, 1 indicates small lesions with 1–25% of the area affected, 2 indicates moderate changes with 26–50% of the area affected, and 3 indicates severe lesions affecting >50% of the area). The total lung injury score is 9 [27,28].

### 2.10. Statistical Analysis

All data are presented as the mean ± standard error of the mean (SEM). Statistical analyses were performed using GraphPad Prism 8 software (GraphPad Software, La Jolla, CA, USA). Differences in the disease activity index (DAI) between the ND and TD groups before intratracheal instillation of saline or LPS were analyzed using an unpaired Student’s *t*-test. For outcomes measured after intratracheal instillation, differences among the four DSS-treated groups, namely ND-Sal, ND-LPS, TD-Sal, and TD-LPS, were analyzed using the two-way analysis of variance (ANOVA). The two factors were diet treatment (ND vs. TD) and intratracheal challenge (saline vs. LPS). When significant main effects or interactions were detected, Bonferroni’s post hoc test was performed for multiple comparisons. A *p*-value of < 0.05 was considered statistically significant. Power estimations were performed for ANOVA based on Cohen’s f effect size using representative acute lung injury outcomes, including BALF neutrophil percentage, lung injury score, and lung oxidative stress [29].

## 3. Results

### 3.1. DAI of the DSS-Treated Groups

**Figure 1:**
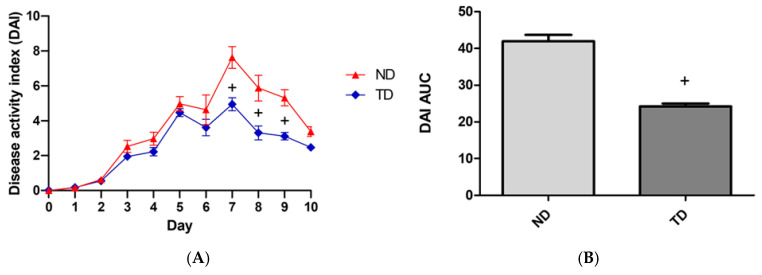
(**A**) Disease activity index (DAI) and (**B**) area under the curve (AUC) of the dextran sodium sulfate (DSS)-treated groups with normal or tryptophan (Try)-containing diets during the experimental period. ND, normal diet with DSS-induced colitis; TD, Try diet with DSS-induced colitis (*n* = 8 for each group). Values are expressed as the mean ± SEM. Differences between the two DSS-treated groups were analyzed through a *t*-test. ^+^ Significantly differs from the TD group (*p* < 0.05).

Mice fed a normal diet had a higher DAI at days 7–9 and AUC than those fed the Try-containing diet when exposed to DSS and during the recovery period (Figure 1).

### 3.2. Histopathological Findings of the Colon in DSS-Treated Groups with Intratracheal Instillation

**Figure 2:**
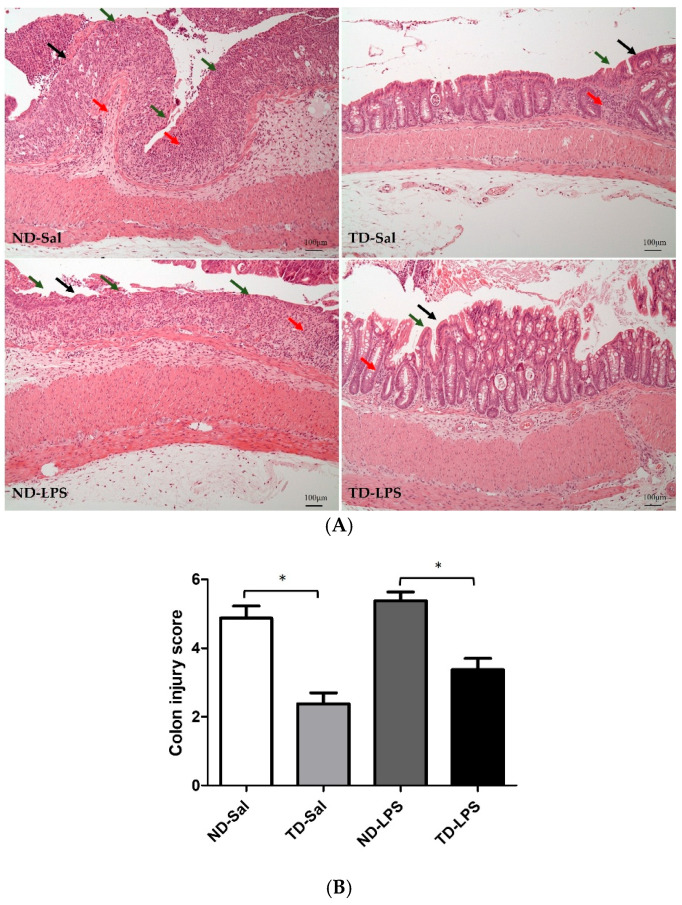
(**A**) Representative histological images of colon sections stained with hematoxylin and eosin (H&E) and (**B**) colon injury scores in dextran sodium sulfate (DSS)-treated groups. Green arrows indicate loss of epithelium; black arrows indicate epithelial injury/ulceration; red arrows indicate inflammatory cell infiltration. ND-Sal, DSS-treated group with a normal diet and intratracheal instillation of saline; TD-Sal, DSS-treated group with a Try diet and intratracheal instillation of saline; ND-LPS, DSS-treated group with a normal diet and intratracheal instillation of LPS; TD-LPS, DSS-treated group with a Try diet and intratracheal instillation of LPS (*n* = 8 for each group). Values are expressed as the mean ± SEM. Differences among the four DSS-treated groups were analyzed through the two-way analysis of variance (ANOVA) followed by the Bonferroni post hoc test. Statistical significance between the indicated groups is shown by brackets. * *p* < 0.05.

Findings showed that loss of epithelium, inflammatory cell infiltration, and epithelial injury/ulceration were more severe in the ND-Sal group than in the TD-Sal group (Figure 2). Similar pathological changes were also observed after intratracheal LPS instillation, with greater colonic damage in the ND-LPS group than in the TD-LPS group. These findings suggest that dietary tryptophan supplementation attenuated DSS-associated colonic histological injury, and a similar protective trend was maintained after intratracheal LPS challenge in this experimental setting (Figure 2).

### 3.3. Plasma LPS and Cytokine Concentrations in DSS-Treated Groups with Intratracheal Instillation

**Figure 3:**
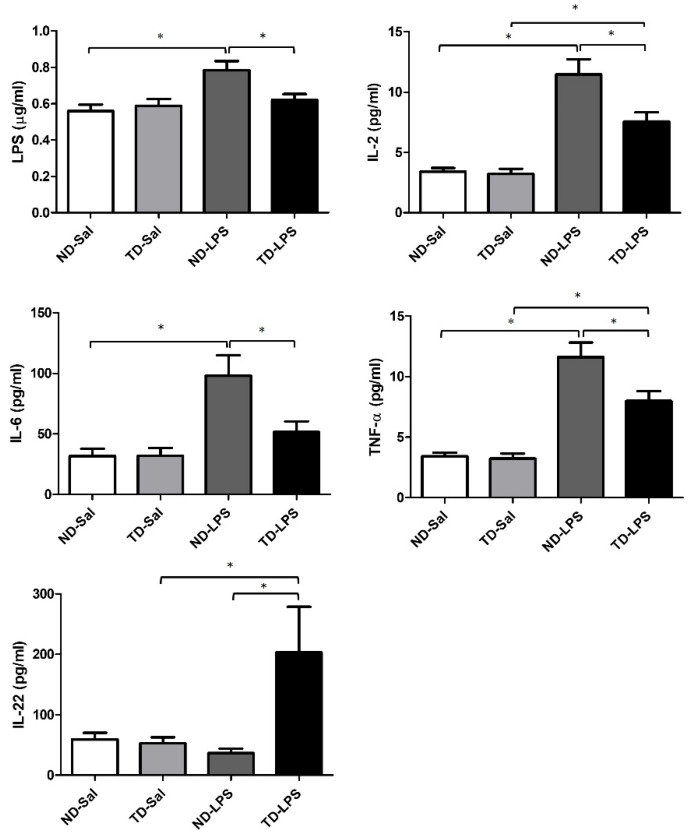
Plasma lipopolysaccharide (LPS) and cytokine levels in dextran sodium sulfate (DSS)-treated groups with intratracheal saline or LPS instillation. Plasma levels of LPS, interleukin (IL)-2, IL-6, tumor necrosis factor (TNF)-α, and IL-22 were measured in the ND-Sal, TD-Sal, ND-LPS, and TD-LPS groups. ND-Sal, DSS-treated group fed a normal diet with intratracheal instillation of saline; TD-Sal, DSS-treated group fed a tryptophan (Try)-supplemented diet with intratracheal instillation of saline; ND-LPS, DSS-treated group fed a normal diet with intratracheal instillation of LPS; TD-LPS, DSS-treated group fed a Try-supplemented diet with intratracheal instillation of LPS (*n* = 8 per group). Values are expressed as the mean ± SEM. Differences among the four DSS-treated groups were analyzed using the two-way analysis of variance (ANOVA), followed by the Bonferroni post hoc test. Statistical significance between the indicated groups is shown by brackets. * *p* < 0.05.

There were no differences in plasma LPS levels between the two DSS-treated groups with saline instillation. Intratracheal LPS stimulation significantly increased the plasma LPS concentration. Also, IL-2, IL-6, and TNF-α levels were elevated. The group with Try supplementation exhibited significantly reduced LPS, IL-2, IL-6, and TNF-α levels, whereas IL-22 levels increased after LPS instillation (Figure 3).

### 3.4. Concentrations of Proinflammatory Cytokines and Chemotactic Factors in BALF of DSS-Treated Groups

**Figure 4:**
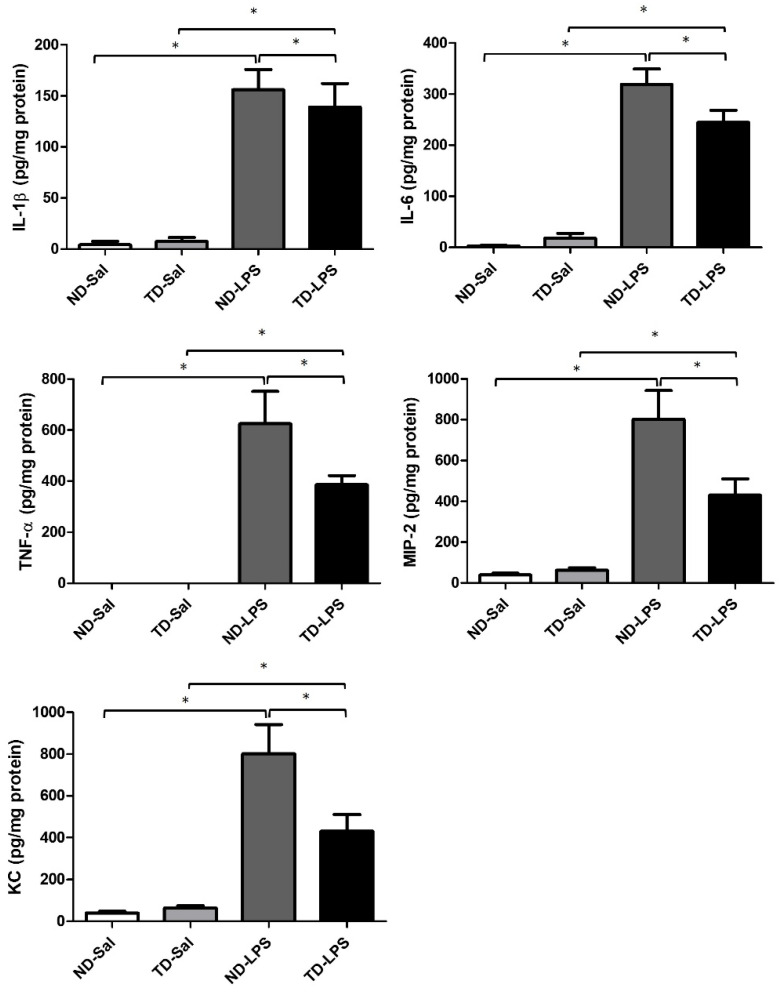
Concentrations of proinflammatory cytokines and chemotactic factors in bronchoalveolar lavage fluid (BALF) of dextran sodium sulfate (DSS)-treated groups with intratracheal saline or lipopolysaccharide (LPS) instillation. BALF levels of interleukin (IL)-1β, IL-6, tumor necrosis factor (TNF)-α, macrophage inflammatory protein (MIP)-2, and keratinocyte-derived chemokine (KC) were measured in the ND-Sal, TD-Sal, ND-LPS, and TD-LPS groups. ND-Sal, DSS-treated group fed a normal diet with intratracheal instillation of saline; TD-Sal, DSS-treated group fed a tryptophan (Try)-supplemented diet with intratracheal instillation of saline; ND-LPS, DSS-treated group fed a normal diet with intratracheal instillation of LPS; TD-LPS, DSS-treated group fed a Try-supplemented diet with intratracheal instillation of LPS (*n* = 8 per group). Values are expressed as the mean ± SEM. Differences among the four DSS-treated groups were analyzed through the two-way analysis of variance (ANOVA) followed by the Bonferroni post hoc test. Statistical significance between the indicated groups is shown by brackets. * *p* < 0.05.

To test the extent of lung inflammation in response to LPS stimulation and Try treatment, inflammatory mediator levels in BALF were measured. We found that LPS instillation led to increments in IL-1β, IL-6, TNF-α, CXCL1/KC, and CXCL2/MIP-2 concentrations. Compared to the saline-injected group, Try administration reduced IL-6 and CXCL2/MIP-2 levels after LPS instillation (Figure 4).

### 3.5. BALF Macrophage and Neutrophil Distributions in DSS-Treated Groups

**Figure 5:**
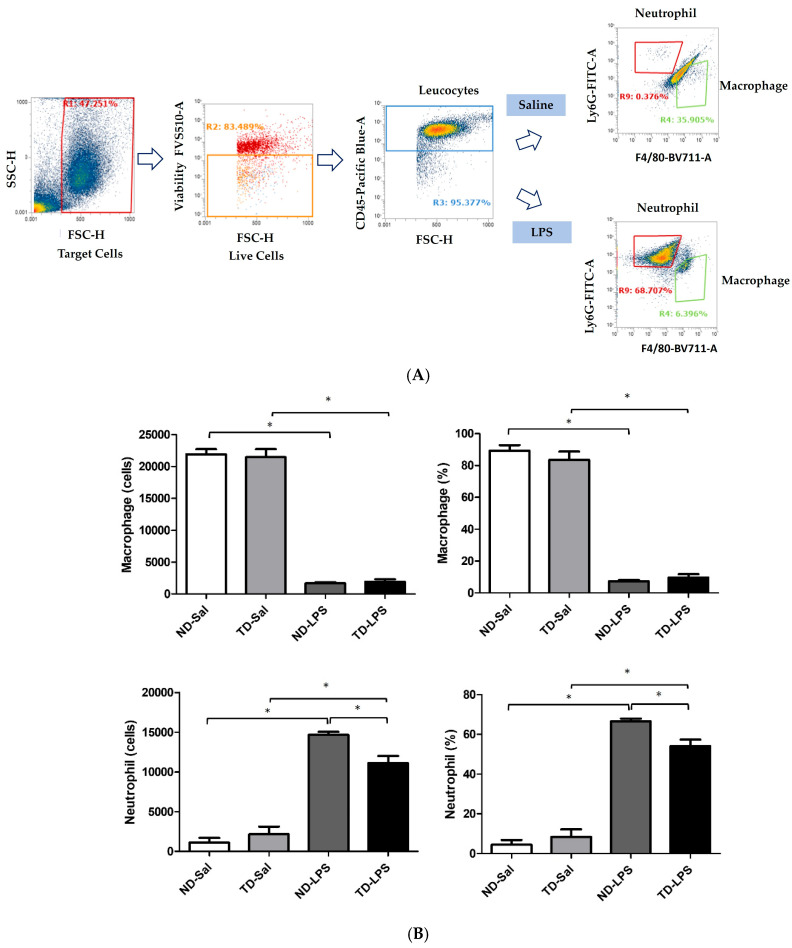
(**A**) Representative flow cytometric gating strategy for bronchoalveolar lavage fluid (BALF) macrophages and neutrophils. (**B**) Total counts and percentages of macrophages and neutrophils in BALF of dextran sodium sulfate (DSS)-treated groups. Macrophages and neutrophils were identified as CD45.2+/F4/80+ and CD45.2+/Ly-6G+ cells, respectively. ND-Sal, DSS-treated group fed a normal diet with intratracheal instillation of saline; TD-Sal, DSS-treated group fed a tryptophan (Try)-supplemented diet with intratracheal instillation of saline; ND-LPS, DSS-treated group fed a normal diet with intratracheal instillation of lipopolysaccharide (LPS); TD-LPS, DSS-treated group fed a Try-supplemented diet with intratracheal instillation of LPS (*n* = 8 per group). Values are expressed as the mean ± SEM. Differences among the four DSS-treated groups were analyzed through the two-way analysis of variance (ANOVA) followed by the Bonferroni post hoc test. Statistical significance between the indicated groups is shown by brackets. * *p* < 0.05.

To determine immune cell infiltration in the lungs following LPS instillation and Try administration, macrophage and neutrophil distributions in BALF were analyzed. Figure 5A shows representative flow cytometric plots of the gating strategy for macrophages and neutrophils. LPS instillation markedly altered the BALF immune cell profile, characterized by increased neutrophil counts and percentages and decreased macrophage counts and percentages compared with the saline-instilled groups (Figure 5B). These findings suggest a shift from a macrophage-predominant BALF cell profile toward neutrophil-dominant pulmonary inflammation after LPS challenge, rather than a direct inhibitory effect of LPS on macrophage accumulation. No significant differences in macrophage counts or percentages were observed between the ND-LPS and TD-LPS groups. In contrast, Try supplementation significantly reduced neutrophil counts and percentages in BALF after LPS instillation.

### 3.6. mRNA Expressions of TLR4 Pathway-Related Factors and Downstream Inflammatory Cytokines in Lung Tissues of the DSS-Treated Groups

**Figure 6:**
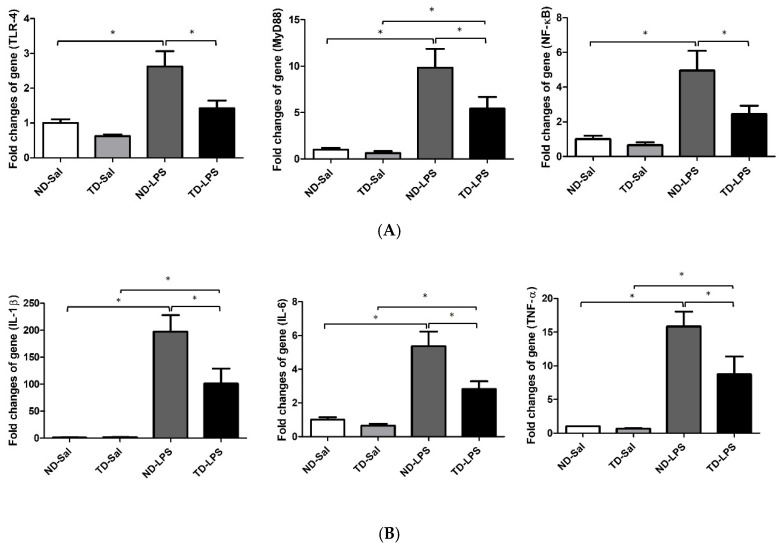
(**A**) mRNA expression of toll-like receptor 4 (TLR4) pathway-related factors. (**B**) mRNA expression of downstream inflammatory cytokines in lung tissues of dextran sodium sulfate (DSS)-treated groups. MyD88, myeloid differentiation factor 88; NF-κB, nuclear factor-κB; IL, interleukin; TNF, tumor necrosis factor. ND-Sal, DSS-treated group fed a normal diet with intratracheal instillation of saline; TD-Sal, DSS-treated group fed a tryptophan (Try)-supplemented diet with intratracheal instillation of saline; ND-LPS, DSS-treated group fed a normal diet with intratracheal instillation of lipopolysaccharide (LPS); TD-LPS, DSS-treated group fed a Try-supplemented diet with intratracheal instillation of LPS (*n* = 8 per group). Values are expressed as the mean ± SEM. Differences among the four DSS-treated groups were analyzed through the two-way analysis of variance (ANOVA) followed by the Bonferroni post hoc test. Statistical significance between the indicated groups is shown by brackets. * *p* < 0.05.

After LPS stimulation, mRNA expressions of *TLR4, MyD88*, and *NF-κB* significantly increased. Compared to the LPS-treated group without Try supplementation, the Try-supplemented group had lower *TLR4*, *MyD88*, and *NF-κB* expressions (Figure 6A). Downstream inflammatory genes, including *IL-1β*, *IL-6*, and *TNF-α*, in the TD group were significantly lower than those of the ND group when LPS was intratracheally instilled (Figure 6B).

### 3.7. mRNA Expressions of AhR/IL-22/IL-22R Signaling and TJ Protein Levels in Lung Tissues of the DSS-Treated Groups

**Figure 7:**
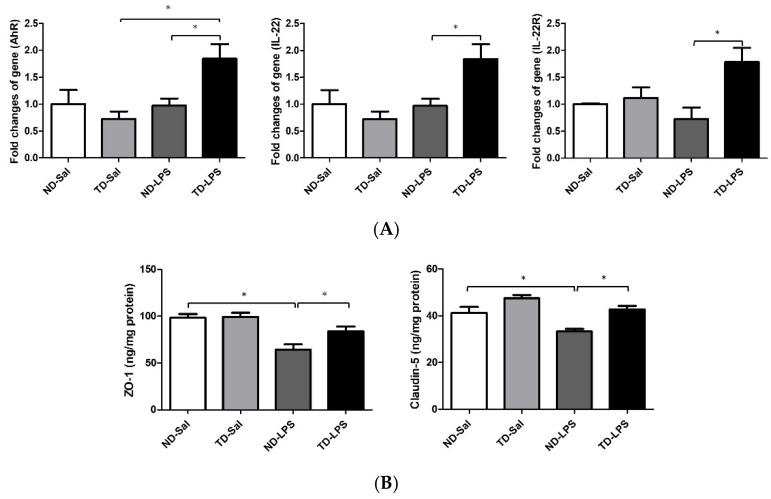
(**A**) Messenger RNA (mRNA) expression of aryl hydrocarbon receptor (AhR)/interleukin-22 (IL-22)/IL-22 receptor (IL-22R) signaling-related genes and (**B**) tight junction protein levels in lung tissues of dextran sodium sulfate (DSS)-treated groups. ND-Sal, DSS-treated group with a normal diet and intratracheal instillation of saline; TD-Sal, DSS-treated group with a Try diet and intratracheal instillation of saline; ND-LPS, DSS-treated group with a normal diet and intratracheal instillation of LPS; TD-LPS, DSS-treated group with a Try diet and intratracheal instillation of LPS (*n* = 8 for each group). Values are expressed as the mean ± SEM. Differences among the four DSS-treated groups were analyzed through the two-way analysis of variance (ANOVA) followed by the Bonferroni post hoc test. Statistical significance between the indicated groups is shown by brackets. * *p* < 0.05.

The Try-supplemented group exhibited significantly enhanced expression levels of *AhR*, *IL-22*, and *IL-22R* compared to those in the group without dietary Try when LPS was intratracheally instilled (Figure 7A). To evaluate whether LPS destroys and Try improves the integrity of the alveolar–capillary barrier, some TJs were analyzed. ZO-1 levels were significantly lower in the LPS-treated ND group than in groups with saline instillation. Also, levels of claudin-5 were lower in the ND group after LPS than the TD group with saline instillation. The DSS-treated group with Try supplementation showed reversals with reduced ZO-1 and claudin-5 levels stimulated by LPS (Figure 7B).

### 3.8. MDA and 4-HNE Concentrations in Lungs of DSS-Treated Groups

**Figure 8:**
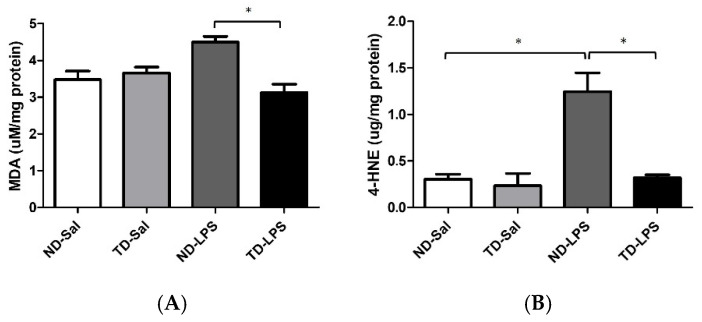
(**A**) Malondialdehyde (MDA) and (**B**) 4-hydroxynonenal (4-HNE) levels in lung tissues of dextran sodium sulfate (DSS)-treated groups. ND-Sal, DSS-treated group with a normal diet and intratracheal instillation of saline; TD-Sal, DSS-treated group with a Try diet and intratracheal instillation of saline; ND-LPS, DSS-treated group with a normal diet and intratracheal instillation of LPS; TD-LPS, DSS-treated group with a Try diet and intratracheal instillation of LPS (*n* = 8 for each group). Values are expressed as the mean ± SEM. Differences among the four DSS-treated groups were analyzed through the two-way analysis of variance (ANOVA) followed by the Bonferroni post hoc test. Statistical significance between the indicated groups is shown by brackets. * *p* < 0.05.

To verify the extent of oxidative stress of lung tissues, MDA and 4-HNE levels were analyzed (Figure 8). The findings showed that elevation of MDA was not obvious, but 4-HNE levels significantly increased after LPS instillation. The TD group exhibited lower MDA and 4-HNE levels than the ND group upon LPS stimulation, and had no difference from the saline-instilled groups.

### 3.9. Histopathological Aspect of the Lungs in DSS-Treated Groups

**Figure 9:**
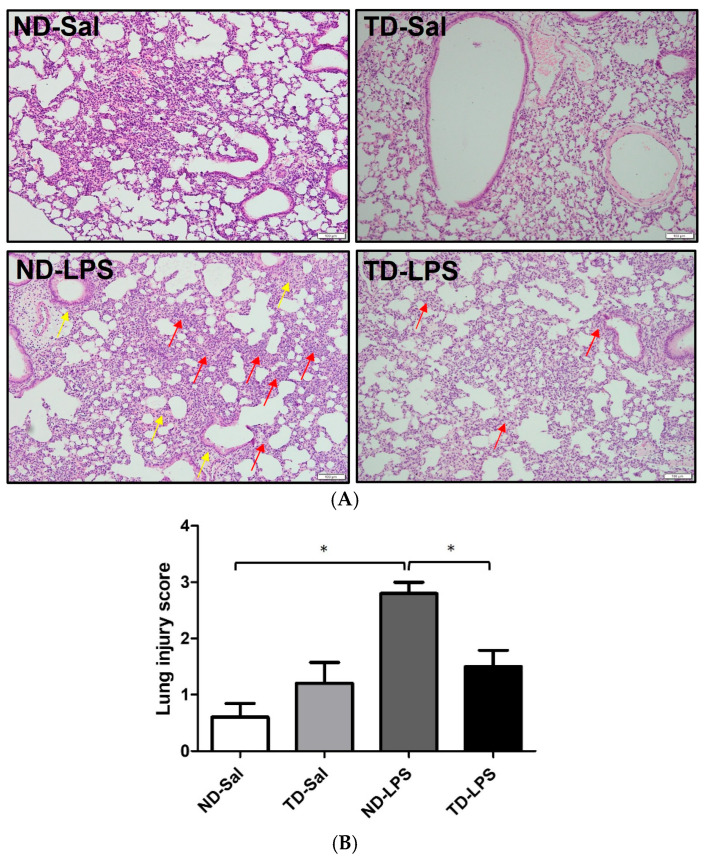
(**A**) Representative histological images of lung sections stained with hematoxylin and eosin (H&E) and (**B**) lung injury scores in dextran sodium sulfate (DSS)-treated groups. Red arrows indicate alveolar wall thickening; yellow arrows indicate perivascular infiltration of inflammatory leukocytes. ND-Sal, DSS-treated group with a normal diet and intratracheal instillation of saline; TD-Sal, DSS-treated group with a Try diet and intratracheal instillation of saline; ND-LPS, DSS-treated group with a normal diet and intratracheal instillation of LPS; TD-LPS, DSS-treated group with a Try diet and intratracheal instillation of LPS (*n* = 8 for each group). Values are expressed as the mean ± SEM. Differences among the four DSS-treated groups were analyzed through the two-way analysis of variance (ANOVA) followed by the Bonferroni post hoc test. Statistical significance between the indicated groups is shown by brackets. * *p* < 0.05.

Findings showed that the alveolar wall thickness, perivascular wall PMN infiltration, and edema were more severe as observed in the ND group than the TD group after LPS instillation (Figure 9). These phenomena were not obviously noted in the saline-instilled groups. Lower injury scores were also found in groups with saline instillation and the Try-supplemented group with LPS instillation.

### 3.10. Power Estimation for Animal Number

Power estimation showed that the calculated Cohen’s f values were 3.66, 1.30, and 1.06 for BALF neutrophil percentage, lung injury score, and lung oxidative stress, respectively. With eight mice per group at a two-sided α level of 0.05, the achieved powers for all three representative outcomes were at least 99%. These findings indicate that eight mice per group were sufficient to detect the observed differences while complying with the 3Rs principle of reduction and avoiding unnecessary animal use.

## 4. Discussion

A previous study demonstrated that a tryptophan-containing diet suppressed colonic inflammation and improved epithelial homeostasis, thereby alleviating DSS-induced acute colitis [16]. Consistent with this finding, dietary tryptophan supplementation reduced DAI and DAI AUC and attenuated DSS-associated colonic histological injury in the present study. In addition, previous evidence has shown that 2% DSS-induced colitis represents a relatively mild inflammatory condition that causes local colonic injury, whereas systemic changes in circulating inflammatory cytokines are less pronounced and become more evident in mice with more severe colitis induced by higher DSS concentrations [30]. Consistent with this evidence, tryptophan supplementation in the present study improved DSS-induced intestinal disease activity, as reflected by reduced DAI, DAI AUC, and colon injury score, but did not substantially alter plasma LPS or inflammatory cytokine levels under the saline-challenged condition. This suggests that circulating inflammatory markers may not fully reflect the local intestinal protective effects of tryptophan in a relatively mild DSS-induced colitis model. In the present study, we further investigated the effects of dietary tryptophan supplementation on LPS-induced pulmonary inflammatory injury under DSS-induced colitis conditions. Our findings indicate that dietary tryptophan supplementation attenuated LPS-induced pulmonary inflammation and oxidative stress, accompanied by reduced systemic inflammatory responses after LPS challenge, improved barrier-related gene expression, and ameliorated histological findings. Increased expression of AhR/IL-22/IL-22R pathway-related genes, together with reduced neutrophil infiltration and downregulation of TLR4/MyD88/NF-κB signaling, suggests that this pathway may be involved in the protective effects of dietary tryptophan supplementation in this experimental setting.

ALI is characterized by dysregulated immune cells and exuberant inflammatory mediator generation [31]. In this study, we analyzed total counts and percentages of macrophages and neutrophils in BALF. Alveolar macrophages are a resident population in a resting condition that can eliminate invading pathogens and maintain pulmonary immune homeostasis [32]. Neutrophils are the most abundant circulating leukocytes and are regarded as the first line of defense in innate immunity [33]. During respiratory infection, macrophages are activated, and mediators released by injured pulmonary tissues attract neutrophils to the site of inflammation [32,33,34]. However, bacterial infections, inflammatory conditions, and reactive species production may lead to apoptosis and programmed necrotic macrophage death [35]. In this study, we did find elevated inflammatory cytokines in BALF and higher lipid peroxide levels in lung tissues. CXCL1/KC and CXCL2/MIP-2 are potent attractants of leukocyte migration into inflamed tissues [36]. The exaggerated inflammation and oxidative stress, and increased CXCL1 and CXCL2 levels were consistent with decreased totals and percentages of macrophages as well as elevated neutrophils in BALF after LPS instillation. Dietary Try supplementation reduced pulmonary neutrophil infiltration and inflammatory cytokine generation in this colitis model when the lungs were insulted with LPS.

LPS is the principle agent in the pathogenesis of endotoxemia, which is triggered by activation of TLR4 expression [37]. Myeloid differentiation factor 88 (MyD88) is an adapter protein involved in signal transduction by TLRs and acts as a link between the receptors and downstream kinases [38]. Once TLR4 is activated, MyD88 is recruited and initiates downstream signaling and the NF-κB pathway. The TLR4/MyD88/NF-κB axis promotes inflammatory mediator production and has important implications for damage to tissues and organs [39,40]. In this study, we found that LPS-instilled DSS groups exhibited elevated expressions of the *TLR4*, *MyD88*, and *NF-κB* genes. Also, mRNA expressions of inflammatory cytokines, including *IL-1β*, *IL-6*, and *TNF-α*, were upregulated in lung tissues, which was consistent with the elevated inflammatory cytokines in BALF. On the contrary, Try alleviated the TLR4-signal pathway by downregulating MyD88- and NF-κB-associated downstream inflammatory cytokine expressions that prevented excessive inflammation stimulated by intratracheal LPS instillation.

Increased pulmonary vascular endothelial cell and alveolar epithelial barrier dysfunction are major pathological features of ALI [41,42]. Neutrophil immigration, and exaggerated inflammation and oxidative stress are factors that result in increased permeability and deterioration of the integrity of the alveolar-capillary barrier [43]. In this study, some selected TJ proteins in lung tissues that constitute and maintain the barrier integrity were measured. ZO-1 contains multiple domains that binds to and organizes other junctional components in maintaining mucosal homeostasis and the barrier function. Claudin-5 is expressed by pulmonary epithelial and endothelial cells that enable tight attachment among cells [44,45,46]. We observed that the DSS-treated group with LPS instillation had lower TJ protein expressions suggesting that the pulmonary barrier integrity had been disrupted. Try supplementation improved the alveolar-capillary barrier integrity in ALI concurrent with colitis.

The potential mechanisms responsible for the favorable effects of a Try-containing diet on alleviating pulmonary inflammation and improving barrier integrity after intratracheal instillation of LPS in colitis mice may be partly attributed to the action of AhR/IL-22/IL-22R signaling. A previous study revealed that AhR combines with signal transducer and activator of transcription 1 (Stat1), which controls the LPS-TLR4-mediated inflammatory response. The AhR-Stat1 complex binds to NF-κB, suppresses its transcriptional activity, and downregulates subsequent inflammatory cytokine generation [8]. Diverse experimental systems have addressed the role of AhR ligands in resolving skin inflammation and osteoarthritis [7,47,48]. In this study, a Try-containing diet significantly upregulated AhR expression and concomitantly suppressed TLR4/MyD88/NF-κB signaling. These findings suggest that AhR-related signaling may be involved in the attenuation of pulmonary inflammation stimulated by LPS instillation concurrent with colitis. In this study, we also showed that Try supplementation improved the integrity of the alveolar-capillary barrier. IL-22 is a cytokine which has multiple roles in maintaining epithelial homeostasis and host defense [49]. Previous studies reported that AhR ligands enhance IL-22 production in intestinal group 3 innate lymphoid cells (ILC3s) and exert their function on regeneration and reconstruction of the intestinal epithelium [50,51]. The interaction of IL-22 with its receptor, IL-22R, drives signaling that provides protection against epithelial injury [52]. In the present study, dietary Try supplementation upregulated AhR/IL-22/IL-22R expression and was associated with alleviated lung barrier injury under LPS instillation. A recent study also revealed that AhR activity enhanced barrier function during lung injury and repair [10]. Since pulmonary inflammation and oxidative stress impair barrier integrity, attenuation of inflammation and reductions in lipid peroxide levels observed in the Try-supplemented group may also be responsible for ameliorating alveolar-capillary barrier damage. The histological findings were consistent with these results, showing that lung injury was attenuated after LPS instillation when Try was given to colitis mice. Based on body surface area conversion, the supplemental tryptophan dose used in this study is estimated to correspond to approximately 4.9 g/day for a 60-kg adult [53]. This amount exceeds the estimated adult nutritional requirement for tryptophan and would be attainable primarily through supplementation rather than typical dietary intake. Therefore, the translational relevance of this dose should be interpreted cautiously. This study has several limitations. First, because DSS induced colitis is a well-established model with known clinical implications, a non-DSS-treated control group was not included. As such, the current design reflects the cumulative effects of both colitis and LPS, rather than attributing outcomes to either condition independently. Second, neither LPS clearance nor functional pulmonary permeability was assessed, the mechanism that TD-LPS group reduced circulating LPS levels required further explored. Third, several mechanistic findings including AhR/IL-22/IL-22R axis and the TLR4/MyD88/NF-κB inflammatory pathway were analyzed primarily on mRNA expression and ELISA. The involvement of these pathways in the tryptophan-supplemented group requires further validation at the protein and signaling-activation levels.

## 5. Conclusions

**Figure 10:**
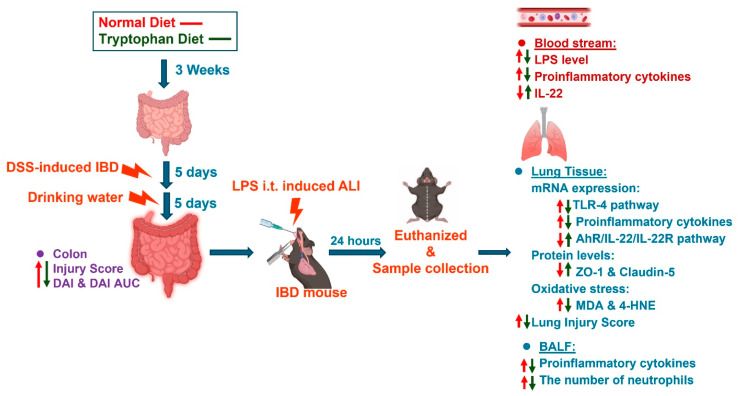
Illustration of the potential mechanisms of tryptophan administration after LPS-induced acute lung injury. AhR: aryl hydrocarbon receptor; BALF: bronchoalveolar lavage fluid; DAI: disease activity index; DAI AUC: area under the curve of disease activity index; DSS: dextran sulfate sodium; IBD: inflammatory bowel disease; IL-22: interleukin-22; IL-22R: interleukin-22 receptor; i.t.: intratracheal injection; LPS: lipopolysaccharide; MDA: malondialdehyde; TLR-4: toll-like receptor 4; ZO-1: Zonula Occludens-1; 4-HNE: 4-hydroxynonenal. (Created in BioRender. Hao, Y.-P. (2026) https://BioRender.com/5vlgr4e).

In summary, our findings indicated that intratracheal LPS instillation in colitis mice led to pulmonary inflammation, neutrophil infiltration, oxidative stress, and impaired barrier integrity (Figure 10). Mice that consumed a Try-containing diet exhibited ameliorated lung inflammation, reduced neutrophil infiltration, an attenuated TLR4 signaling pathway, upregulated AhR/IL-22/IL-22R expressions, and improved barrier integrity that may have subsequently alleviated lung injury. These findings imply that dietary Try supplementation may have potential therapeutic significance in bacterial-induced ALI in a colitis condition.

## Data Availability

The data supporting the findings of this study are available from the corresponding author upon reasonable request.

## Supplementary Materials

The following supporting information can be downloaded at: https://www.mdpi.com/article/10.3390/nu18132042/s1, Table S1: Compositions of the control and the tryptophan-containing diets; Table S2: Sequences of the target mRNA primer.

## Abbreviations

The following abbreviations are used in this manuscript:

4-HNE, 4-hydroxynonenal; AhR, aryl hydrocarbon receptor; ALI, acute lung injury; ARDS, acute respiratory distress syndrome; AUC, area under the curve; BALF, bronchoalveolar lavage fluid; CXCL1, C-X-C motif chemokine ligand 1; DAI, disease activity index; DSS, dextran sulfate sodium; IBD, inflammatory bowel disease; IL, interleukin; ILC3s, group 3 innate lymphoid cells; KC, keratinocyte-derived chemokine; LPS, lipopolysaccharide; MDA, malondialdehyde; MIP-2, macrophage inflammatory protein-2; MyD88, myeloid differentiation primary response 88; NF-κB, nuclear factor kappa B; STAT1, signal transducer and activator of transcription 1; TBARS, thiobarbituric acid-reactive substances; TJ, tight junction; TLR4, toll-like receptor 4; TNF-α, tumor necrosis factor-α; Try, tryptophan; ZO-1, zonula occludens-1.
