# Supplementary material for: Dietary Tryptophan Supplementation Attenuates Lipopolysaccharide-Induced Acute Lung Injury in a Murine Model of Colitis
Source: Nutrients. 2026 Jun 23;18(13):2042. doi: 10.3390/nu18132042 (PMC13363077; doi:10.3390/nu18132042)
Supplement: Supplementary file 1 [file nutrients-18-02042-s001.zip › nutrients-4334958-supplementary.pdf]

Supplementary Table S1. Compositions of the control and the tryptophan-containing diets

Composition	Control diet	Tryptophan diet
Tert-butylhydroquinone	0.008	0.008
L-Cysteine	1.8	1.8
Choline bitartrate	2.5	2.5
Vitamin mixture[1]	10	10
Mineral mixture[2]	35	35
Soybean oil	40	40
Cellulose	50	50
Sucrose	100	100
Casein	140	140
Cornstarch	620.70	615.0
Tryptophan	-	5
Total (g)	1000.008	1000.008

[1] The composition of the mineral mixture was as follows (g/1000 g): potassium citrate, 330; calcium phosphate, 260; calcium carbonate, 110; sodium chloride, 51.8; magnesium sulfate, 51.52; magnesium oxide, 8.38; ferric citrate 4.2; manganese carbohydrate hydrate, 2.45; zinc carbonate, 1.12; chromium potassium sulfate, 0.39; copper carbonate, 0.21; ammonium molybdate tetrahydrate, 0.06; sodium fluoride, 0.04; sodium selenite, 0.01; potassium iodate, 0.01.

[2] The composition of the vitamin mixture was as follows (g/100 g): vitamin E acetate, 10; niacin, 3; biotin (1%), 2; pantothenic acid, 1.6; vitamin D3, 1; vitamin B12, 1; vitamin A acetate, 0.8; pyridoxine HCl, 0.7; riboflavin, 0.6; thiamine HCl, 0.6; folic acid, 0.2; menadione sodium bisulfite, 0.08.

Supplementary Table S2. Sequences of the target mRNA primer.

Gene name	Primer sequence (5′→3′)	Accession no.
AhR	**F:** GGCTTTCAGCAGTCTGATGTC	AF405563.1
	**R:** CATGAAAGAAGCGTTCTCTGG	
IL-1β	**F:** TGCCACCTTTTGACAGTGATG	NM_008361.4
	**R:** ATGTGCTGCTGCGAGATTTG	
IL-6	**F:** TCCTACCCCAATTTCCAATGCTC	NM_012589.1
	**R:** TTGGATGGTCTTGGTCCTTAGCC	
IL-22	**F:** TGCGATCTCTGATGGCTGTC	NM_016971.2
	**R:** GACGATGTATGGCTGCTGGA	
IL22RA1	**F:** CACACCGGTCCTCTCGGAAG	NM_178257.2
	**R:** GGCACTTTCCTTGGACAATATCGG	
MyD88	**F:** CATGGTGGTGGTTGTTTCTGAC	NM_010851.3
	**R:**TGGAGACAGGCTGAGTGCAA	
NF-κB	**F:** TTAGCCAGCGAATCCAGACC	M61909.1
	**R:** AGTTCCGGTTTACTCGGCAG	
TLR4	**F:** AGAAATTCCTGCAGTGGGTCA	NM_021297.3
	**R:** TCTCTACAGGTGTTGCACATGTCA	
TNF-α	**F:** ATGGCCTCCCTCTCATCAGT	NM_013693.3

	**R:** TTTGCTACGACGTGGGCTAC	
18s rRNA	**F:** AGTTCGCTCACACCCGAAAT	AH002077.2
	**R:** AGTGCGTTCGAAGTGTCGAT	

ACE, angiotensin-converting enzyme; AhR, aryl hydrocarbon receptor; AT1R, angiotensin II type I receptor; AT2R, angiotensin II type II receptor; IL, interleukin; MyD, myeloid differentiation primary response; NF-κB, nuclear factor kappa-light-chain-enhancer of activated B cells; TLR, toll-like receptor; TNF-α, tumor necrosis factor-α; 18s rRNA, 18S ribosomal RNA; F, forward; R, reverse.
